# A New Approach to Evaluating the Risk–Benefit Equation for Dual-Use and Gain-of-Function Research of Concern

**DOI:** 10.3389/fbioe.2018.00021

**Published:** 2018-03-08

**Authors:** Michael J. Imperiale, Arturo Casadevall

**Affiliations:** ^1^Department of Microbiology and Immunology, University of Michigan, Ann Arbor, MI, United States; ^2^Department of Molecular Microbiology and Immunology, Johns Hopkins School of Public Health, Baltimore, MD, United States

**Keywords:** dual-use research, biosafety, biosecurity policy, research ethics, pandemics

## Abstract

In the twenty-first century, biology faces a problem that has previously vexed other disciplines such as physics, namely the prospect that its knowledge domain could be used to generate biological agents with altered properties that enhanced their weapon potential. Biological weapons bring the additional dimension that these could be self-replicating, easy to manufacture and synthesized with commonly available expertise. This resulted in increasing concern about the type of research done and communicated, despite the fact that such research often has direct societal benefits, bringing the dual-use dilemma to biology. The conundrum of dual use research of concern was crystallized by the so-called “gain-of-function” type of experiments in which avian influenza viruses were endowed with new properties in the laboratory such as increased virulence and the capacity for mammalian transmission. After more than a decade of intensive discussion and controversy involving biological experiments with dual-use potential, there is no consensus on the issue except for the need to carry out such experiments in the safest conditions possible. In this essay, we review the topic with the hindsight of several years and suggest that instead of prescribing prohibitions and experimental limitations the focus should be on the importance of scientific questions at hand. We posit that the importance of a scientific question for medical and scientific progress provides a benchmark to determine the acceptable level of risk in biological experimentation.

## Introduction

The use of technology to gain an advantage in human conflict is ancient. Iron replaced bronze in weapons and history is replete with examples of how scientific and technological advances were adapted for war and the acquisition of power. In fact, all technologies are potentially dual use. This trend accelerated with the expansion of knowledge following the scientific and industrial revolutions and affected primarily the disciplines of physics and chemistry, domains of knowledge containing information that was then immediately useful in warfare. The application of biological knowledge to conflict came later even though biological warfare also has a long history. For example, the black death that devastated medieval European society may have begun by an act of biological warfare during the siege of Caffa in 1346 (Wheelis, 2002). British settlers to North America brought smallpox-infested blankets as “gifts” to Native Americans (Duffy, 1951). However, it was not until the molecular biology revolution in the late twentieth century that the potential of biology, and in particular microbiology, in biological warfare and terrorism came into focus. These fears came true with a single act of terrorism involving the mailing of anthrax spores in the U.S. in 2001, which caused several deaths and considerable disruption to government facilities such as congressional offices and the postal system (Bush and Perez, 2012). This act of bioterrorism catalyzed a series of events that led to greater awareness of the potential of using biological knowledge in nefarious ways, new regulations in biological research in the U.S. such as the Select Agents and Toxins list, and heighted concerns about some types of microbiology research.

One of the immediate effects of the post-2001 environment was increased scrutiny of published findings, and several papers were particularly noteworthy for eliciting discussion and concern. In 2001, researchers in Australia showed that insertion of the gene for IL-4 into the ectromelia virus genome defeated vaccine immunity (Jackson et al., 2001), a report that drew immediate concerns because of its possible implication for similar effects with variola major virus. Similarly, the finding that complement inhibition potentiated variola virus virulence suggested a mechanism for enhancing its pathogenicity (Rosengard et al., 2002). An analysis of the U.S. milk distribution system revealed vulnerable nodes where the introduction of botulinum toxin could lead to mass casualties (Wein and Liu, 2005), leading to an outcry about journals publishing research that could be exploited by terrorists. Advances in molecular biology led to the chemical synthesis of poliovirus (Cello et al., 2002) and the reconstruction of the 1918 pandemic influenza virus (Tumpey et al., 2005), which greatly heightened concern about the re-introduction of extinct, controlled, and new viruses to naïve human populations. In this environment, the U.S. Government created the National Science Advisory Board for Biosecurity (NSABB) in 2005 to provide guidance on issues relating to biological risk and security. Both authors of this article were inaugural members of the NSABB and served on that board for 9 years.

When the NSABB first began their deliberations, one of the major early topics was that of dual-use research in the biological sciences. This issue had previously vexed the physics community in the twentieth century as advances in physics and associated technologies had led to radar, nuclear weapons, ballistic missiles, and other military hardware. However, there was little or no precedent for similar concerns in the biological research community, especially since biological weapons were outlawed by the Biological Weapons Convention in 1972. Moreover, the term dual use, which had meant civilian and military use in the physics arena, was redefined in its application to biology by an NRC report in 2004 to mean technologies and information that had both beneficial and maleficent use (NR Council, 2004). Nevertheless, among the early accomplishments of the NSABB was formulating a definition for dual use research of concern (DURC), which limited the scope of worrisome work to a small subset of biological research. The NSABB also generated a set of recommendations for communicating DURC in publications and other venues. The work of the NSABB proceeded quietly and in relative obscurity until 2011 when the U.S. government learned about two submitted manuscripts reporting that highly pathogenic avian influenza virus (HPAIV) could be made mammalian transmissible by laboratory passage in ferrets, and sought an opinion from the board as to whether publication should be permitted.

### The Great Gain-of-Function (GOF) Controversy

Before considering the GOF controversy involving influenza virus, it is worthwhile reviewing what is meant by “GOF” and the limitations of the lexicon in the controversy. At the most basic level, a GOF experiment, as the name implies, is one that gives a biological entity any new property. GOF experiments can be highly beneficial and can generate pest resistant crops, microbes expressing proteins for medical therapy such as recombinant human insulin, and new cancer therapies by enhancing lymphocyte function. The controversial aspects of GOF experiments in microbiology arise when microbes are modified to have new properties that can enhance their virulence and transmissibility because such experiments raise worries about new diseases, outbreaks, pandemics, biosafety, and biosecurity. In the experience of the authors, the NSABB probably did not anticipate what a problematic dual-use paper would look like until they had the opportunity to consider the two papers that reported the gain of mammalian transmissibility for HPAIV (Herfst et al., 2012; Imai et al., 2012). What made the two papers highly controversial was that they reported that only a few amino acid changes were sufficient to confer mammalian transmissibility to the H5N1 HPAIV, and there was concern that this information could be used to generate a virus capable of pandemic potential with high mortality by anyone with basic molecular biology and virology skills. The NSABB initially recommended redacting the sequence information, but this was considered not feasible given the legal framework in the U.S., and the two papers (Herfst et al., 2012; Imai et al., 2012) were eventually published in 2012 after some modifications to the text. The arguments, counterarguments, and events surrounding that episode are described in various publications (Berns et al., 2012a,b; Fouchier et al., 2012a), often by the participants themselves, and will not be repeated here. The major outcome of the great GOF controversy of 2012 is that it defined and crystallized some of the issues of dual-use research in biology by providing clear examples of experiments that were of great scientific value while also raising biosecurity and biosafety concerns. Although both papers were eventually published with full information, the debate and controversy did not solve the central questions of what work should be performed moving forward and how it should be reported. In fact, the controversy erupted again in the summer of 2014 following the publication of additional papers describing other GOF experiments (e.g., Watanabe et al., 2014) as well as a series of biosafety lapses at U.S. government laboratories, which led the government to impose a pause on these types of experiments with certain viruses until the NSABB could formulate new recommendations and guidelines. The pause was lifted in mid-December, 2017, alongside policy guidance about how GOF funding decisions should be made going forward.^1^

### Publication Quandaries

One of the most vexing questions of the problem of dual-use research is publication of scientific findings. In the U.S., the research community is guided by National Security Decision Directive (NSDD) 189, formulated in 1985 by the Reagan administration, which states that all fundamental research results could be published freely. In other words, there was either open publication or classification, and nothing in between. Viewed from the context of NSDD-189 the papers involved in the 2012 GOF controversy were either to be published in their entirety or classified, with the latter alternative being impractical and unfeasible since the work had been carried out in an unclassified environment. With this binary policy, redacting papers because they might contain information useful for nefarious purposes is not an option unless export control regulations are invoked [for a full discussion of the legalities involved, see NAS EM DURC report (National Academies of Sciences Engineering and Medicine, 2017)]. Consequently, when faced with GOF papers containing information that could conceivably be used to enhance the pathogenicity or transmissibility of a virus, editors and journals have almost always opted for full publication, usually requiring more details from the authors about biosafety and biosecurity methods, and often publishing an accompanying editorial emphasizing the scientifically useful aspects of the research [for examples, see Dermody et al. (2013, 2014a)]. Such decisions have sometimes elicited strong criticism from members of the virology community (Dermody et al., 2014b; Wain-Hobson, 2014a).

A more recent situation illustrates the difficult issues faced by authors, editors, and journals when publishing papers that contain DURC. In 2013, the *Journal of Infectious Disease* faced the quandary of receiving a paper reporting a new botulinum toxin that was resistant to neutralization by the then-available antisera. In response, the journal took the remarkable decision of publishing the paper without the toxin sequence and vowed to keep the data confidential until an antidote was available (Casadevall et al., 2013; Dover et al., 2013; Relman, 2013; Hooper and Hirsch, 2014). A major factor in withholding the sequence data was the realization that this toxin could be easily produced using standard techniques if the toxin sequence was available and that the availability of such a toxin might pose a major public health risk. This decision was highly controversial since without the sequence information other investigators could not verify the findings reported in the publication or begin to develop medical countermeasures. In fact, subsequent work revealed that the new toxin was indeed neutralized by available sera, thus diminishing the initial concerns (Enserink, 2015). On one hand, withholding the sequence information from public scrutiny was the responsible action given the potential threat, while on the other hand, such an action delayed verification of the findings and the generation of countermeasures had the threat been real. Clearly, manuscripts containing DURC pose vexing problems for journals that often must make such decisions alone. At American Society for Microbiology journals, there are protocols in place for reviewing such manuscripts and the journals have available many individuals with microbiological and safety expertise who can provide input (Casadevall et al., 2015). Other situations are likely to pose different challenges, but it is clear that journals are often poorly equipped to handle the difficult problems involved in publishing DURC content. In this regard, we have argued for the need of a national board that can help journals make publication decisions (Casadevall et al., 2014), although to date no such body exists.

Another major challenge to the control of DURC information is the emergence of preprint servers in biology that allow the posting of research findings before peer review and the proliferation of predatory open access journals that will publish essentially any paper for a fee. Consequently, there now exist alternative systems for publication even if standard journals decline to publish a particular article over DURC concerns. Bypassing traditional publication methods could also allow authors to avoid government scrutiny.

### The Situation Today

It is not an exaggeration that the situation today regarding DURC and GOF experiments remains highly unsatisfactory. Experimental work involving GOF experiments on pathogens with pandemic potential is highly regulated with proponents of such work arguing that restrictions on experimental design are unwarranted (Fouchier et al., 2012b) while opponents of such experiments argue that such work cannot be justified (Wain-Hobson, 2014a,b). Proponents of GOF experiments have noted that data generated in such experiments is useful in epidemiological surveillance (Schultz-Cherry et al., 2014), whereas opponents have argued that such work cannot be morally justified because of its inherent risk (Lipsitch and Galvani, 2014). In the U.S., government-mandated pauses on certain types of experiments remain in effect for federally funded research but such prohibitions do not extend to other countries, or to work funded by other sources. Although the NSABB continues to analyze and debate the issues involved with DURC and GOF experiments in the U.S., there is no comparable body on the international scene, where much of this type of research is done.

There is no consensus in the scientific community on whether the value of some experiments justifies the risks involved. The central problem in finding a consensus is that the value of the research cannot be measured in real time, while assessments of risk involve assumptions that can lead to widely divergent estimates. For example, some risk–benefit calculations have suggested that a high consequence accident is likely to occur in the near future (Lipsitch and Bloom, 2012; Lipsitch and Inglesby, 2014), while others have estimated such probabilities to be near zero (Fouchier, 2015). However, there is some evidence that the incessant debate, which shows no sign of resolution, is taking its toll on the virology community as shown by surveys suggesting that scientists in training may avoid these areas of research (Pfeiffer, 2015). This is of particular concern given that recent years have seen new viral threats in the forms of the West Africa Ebola outbreak, the emergence of Zika virus in the Americas, and Middle East Respiratory syndrome coronavirus in the Arabian peninsula, highlighting the importance of virology in human preparedness against pandemic threats (Imperiale and Casadevall, 2015a, 2016).

One of the most important developments in the past few decades has been a change in the zeitgeist of the field that concerns itself with DURC research and GOF experiments. In the early years of the twenty-first century, as the U.S. reeled from the 9/11 terrorist attacks, which were shortly followed by the anthrax spore attacks, the focus was primarily on biosecurity. At that time, the concern was that terrorists and state actors would use the tools of the molecular biology revolution to create new and more devastating biological weapons. However, as the years passed and no new attacks developed, combined with the attribution of the anthrax spores in mail attacks to a lone insider in a U.S. government laboratory (Bhattacharjee and Enserink, 2008), this security threat appeared to recede. At the same time, a series of unfortunate biosafety lapses in government laboratories heightened concerns about biosafety. Hence, today people (at least those outside the security community) appear to be more worried about an unintended release of a pathogen with pandemic potential than a deliberate biological attack. This change in emphasis from biosecurity to biosafety has developed slowly and could easily change if there is another deliberate biological attack or if becomes apparent that adversaries are developing biological weapons.

A major problem contributing to the unsatisfactory nature of the current situation is that the NSABB definition of DURC does not work well in day-to-day practice. The NSABB defined DURC as “life sciences research that, based on current understanding, can be reasonably anticipated to provide knowledge, information, products, or technologies that could be directly misapplied to pose a significant threat with broad potential consequences to public health and safety, agricultural crops and other plants, animals, the environment, materiel, or national security.” Although the crafting of this definition was a major achievement at the time in being able to limit the scope of DURC to a relatively small portion of biomedical research, thus leaving most biological research to proceed unfettered, subsequent experience showed that the clause “reasonably anticipated” is so subjective in nature that it requires a risk assessment beyond the capabilities of most scientists, reviewers, and editors (Casadevall et al., 2014). In essence, the definition was helpful in keeping whole swaths of biological research outside the DURC category, such as cancer and immunology research, but it difficult to apply in determining what *is* included in the DURC definition.

Also contributing to an unsatisfactory situation are concerns whether such regulations as the Select Agents and Toxins list help or hinder societal security. On one hand, placing great restrictions on the accessibility to a number of agents and toxins does increase security by making them more difficult to obtain, with the caveat that these are naturally occurring agents that could be obtained from nature by a determined actor. On the other hand, there is the concern that focusing on lists and a relatively short list of agents means that the overwhelming majority of microbial threats are not on the DURC radar screen. For example, Zika virus emerged as a pathogen of pandemic potential with little or no anticipation from experts. Hence, making lists and focusing attention on those agents in the list has the paradoxical potential to reduce biosecurity since regulations are tightened for listed agents, possibly hindering research, while other dangerous agents are neglected (Casadevall and Relman, 2010). A similar argument applies to the current DURC oversight policy of the U.S. government, which is focused on a specific list of agents.^2^ This process, which is akin to searching under the lamppost, carries great danger because it ignores what could be significant threats. In this regard, it is noteworthy that fungal pathogens are seldom considered as biological threats despite the fact that members of this kingdom have significant weapon potential (Casadevall and Pirofski, 2006; Casadevall, 2017).

Against this backdrop of dissatisfaction is the fact that science continues to progress very rapidly, introducing new technologies such CRISPR/Cas9, gene drives, and more efficient synthetic biology, each of which brings with it new possibilities for research that improves the human condition as well as new tools for nefarious purposes. Furthermore, as the technologies improve they are increasingly accessible to more individuals and countries for whom this type of research was previously beyond reach. Hence, the problem of DURC is likely to become significantly more urgent in the near future.

### The Way Forward

In a world where new infectious agents that can rapidly spread among vulnerable populations are described on a regular basis, humanity needs a healthy research establishment focused on microbial threats. It is estimated that there are a minimum of 320,000 mammalian viruses (Anthony et al., 2013): some fraction of these are probably zoonotic events waiting to happen. As Ian Malcolm, the fictional character created by Michael Crichton in the novel *Jurassic Park*, stated, “Life finds a way.” Information and knowledge are the best defenses against these threats. In addition to terror from nature, a new crisis will almost certainly occur in the future arising from a deliberate attack, a new provocative paper, or another biosafety lapse. GOF experiments and DURC research are essential for preparedness and the question is not whether this research should be pursued but rather how to do it safely and mitigate risk. To date, each of the controversies has been reactive, with proponents and opponents of such experiments responding to a specific finding or study. After more than a decade of discussion on what constitutes DURC, benefits and risks of GOF experiments, regulations, pauses, and moratoriums, it is increasingly apparent that current approaches are inadequate for the challenges at hand. Given these limitations, we have proposed a new framework for DURC research that focuses on answering specific questions (Imperiale and Casadevall, 2015b). Hence, instead of prohibiting certain types of experimentation, we suggest that the way forward is to focus on specific scientific questions and the problems that need answers. For example, if there is a need to determine whether a particular feral virus pathogen has the capacity for mammalian infection and transmission, then GOF experiments performed in safe and controlled conditions can be justified. On the other hand, endowing HIV with new transmission properties is not a medically important GOF experiment (National Academies of Sciences Engineering and Medicine, 2017). All human activities that involve probing the unknown, ranging from space exploration to tissue culture procedures, carry some degree of risk, and it is nature of humanity to accept risk to attain progress. Opponents and proponents of this type of experimentation need to maintain open channels for continued discussion because the dialectic of ideas is likely to result in better decisions. Institutional bodies such as the NSABB need to be supported for they constitute important venues for such discussion and recommendations that mitigate risk. In fact, it is important to create similar institutions that can work at the international level since U.S.-based research is a small portion of all microbiological work done worldwide. Most importantly, we should remain optimistic that the research community can do the research necessary to obtain information critical to protect our species while minimizing the risks of such work.

## Author Contributions

Both authors contributed to the writing of this manuscript.

## Conflict of Interest Statement

The authors declare that the research was conducted in the absence of any commercial or financial relationships that could be construed as a potential conflict of interest.

## Footnotes

^1^https://grants.nih.gov/grants/guide/notice-files/NOT-OD-17-071.html.

^2^https://www.phe.gov/s3/dualuse/documents/us-policy-durc-032812.pdf.
